# Pandemic and new perspectives on living: the role of the smart home

**DOI:** 10.3389/fsoc.2023.1243411

**Published:** 2023-11-23

**Authors:** Marina Ciampi, Melissa Sessa

**Affiliations:** ^1^Social Sciences and Economics Department, Sapienza University, Rome, Italy; ^2^Political Sciences Department, Sapienza University, Rome, Italy

**Keywords:** pandemic, living, risk, smart home, sustainability

## Abstract

Based on ongoing multidisciplinary research, this essay offers some theoretical and scenario considerations on the transformations of social rituals in housing contexts during the pandemic period. The analysis focuses on living being not only understood as a “private” experience, but also as a phenomenon of collective interest, especially in relation to issues concerning health emergencies, risk perception, and forms of sociability. In the face of such problems, the sociological perspective has shown its usefulness in providing suitable tools to study the ambivalent and exceptional aspects to which living was exposed during the lockdown period and in the immediate aftermath. Thus, it was chosen to focus attention on the phenomenon of the smart home, an “agent subject,” albeit inanimate, of the process of technological transformation of the housing unit, which can be evaluated not only on the level of environmental sustainability, but also on that of social sustainability.

## 1 The study of the pandemic: sociological perspectives

Since its sudden irruption, the COVID-19 pandemic has presented the characteristics of a “global social fact” that is capable, with its virulence, of radically transforming the face of the 21st century, both on an extensive (global) level and in intensive terms, on different planes of human existence. Inevitably, a phenomenon of such magnitude has mobilized the attention of sociologists for the following reasons: the epistemological variety, research problems, and perspectives of analysis triggered in the socio-cultural, health, ethical, political-economic, and techno-scientific spheres (Bontempi et al., 2020). Like any crisis, the pandemic has operated in our world as a deflagrating force: it has exploded latent tensions in the social, political, and legal magma, forced a rethinking of its paradigms of reference, including constitutional ones, and challenged the very foundations of civil living. The analysis that will be presented in this essay, based on multidisciplinary research, will offer theoretical reflections on the scenario in which the pandemic has transformed social rituals in living contexts. Faced with these problems, the perspective offered by sociology helps to provide tools to try to understand what changes are and have been taking place. In this sense, in the following paragraphs, attention will be paid, on the one hand, to the study of the dynamics triggered by the social actor in the “internal/external” game with the pandemic, and, on the other hand, to the inanimate dynamics, although of equal importance, with the smart home as a vehicle for social sustainability.

In the immediate term, driven by the exceptional nature of the event, sociological studies attempted to answer cognitive questions with different methods: data were constructed and deconstructed, and statistical models or narrative material were used (Galantino, 2020), in a constant virtuous circle between theory and empirical practice. Social scientists, involved as “subjects and objects of study,” questioned the changes, dynamics, and imminent and long-term effects that the pandemic could generate (Morin, 2020; Favretto et al., 2021; Habermas, 2022). They addressed the question of whether the pandemic represented a critical juncture capable of reconfiguring the behaviors of individuals in their interrelationships, the modes of intervention of public institutions, or more generally transforming the very nature of social ties. Based then on the direct experience of a global health risk, the sociologist has directed their gaze to the most radical effects found in the various spheres affected by the crisis. Not marginal in this work is the role of the sociological imagination as a critical tool capable of bringing out dynamics and processes through method and epistemological reflexivity (Wright Mills, 1959) against the simplifying narratives of common sense.

The issues raised immediately appeared not to be confined to the topic of health (and, by extension, to that of life and death) but pertaining to a plurality of aspects: the processes of globalization (Sassen, 2007) and trust in expert systems (Giddens, 1990); the relationship between risk, progress, and science (Beck, 1992, 2016; Luhmann, 1996, 2002); the reproduction of old inequalities (including gender inequalities) and the rise of new socioeconomic fragilities (Sassen, 2014; Mackenbach, 2019; Viviani 2021); the development of new modes of social interaction shaped by social media and digital platforms (Boccia Artieri et al., 2017); “social” distance (Fuchs, 2020) and the rise of community forms deprived of physical co-presence (Bauman, 2014); and the pervasiveness of institutional power in imposing surveillance processes and “historical” measures of spatial confinement to reduce morbidity and mortality (McNeill, 2020).

In this interconnected set of issues, recalled here only as an example and certainly not exhaustive of the entire panorama of studies, the theme of risk has assumed a crucial role because of its obvious ambivalence: although modernity has made it a “calculable factor”—corresponding to the estimate that a given event may come true—it presents itself today as a condition with which to deal with (on an individual and collective level), even if the strategies created to contain its aspects of aleatoriness often contribute to exacerbating its perception as a threat. Indeed, the emergence, thanks in part to the processes of globalization, of a society increasingly exposed to risk entails the acceptance of a common human condition, what Beck (1992) calls the fate of risk: individuals, from birth and despite their best efforts, cannot escape it and all end up relating to it, regardless of class or territorial boundaries. This means that risk has lost the circumscribed character within which early-modern rationality had enclosed it,^1^ moving from international boundaries to the local dimension and feeding on the crises and fragilities of specific local contexts, whether political-institutional, economic, or health-related.

In an attempt to manage the spread of the COVID-19 pandemic under emergency conditions, policymakers in affected countries implemented choices that proved divisive between politics and science, expert knowledge and political classes, and technocracy and democracy (Viviani, 2021). Meanwhile, the virus thrived through human contact and confronted nation-states with choices that compromised normal human sociality to protect public health first and foremost. The legitimization and imposition of social action geared toward the rigidity of confinement highlighted the difficult combination of *immunitas* and *communitas* (Esposito, 2022), that is, between the need for protection/immunization (in defense of biological life) and the desire for community (in defense of relational life). The virus has evaporated the us/you, in/out opposition, bringing to light the inviolable connective infrastructure of social life (Giaccardi and Magatti, 2020). Isolation, the restriction of interpersonal relations, and the reduction of collective freedom, especially in the lockdown phases, have been the necessary responses from a securitarian and control perspective, but they have shown how much the deep meaning of existence lies in openness to the outside, in reciprocity, and in the relationship with the other. It is not possible to think of ourselves as individual islands (Giaccardi and Magatti, 2020, p. 59) because it is in relationship with others, in exchange and dialogue, and in relationship with the biological and technological environment that the social individual is formed. The temporary detachment of the individual from his or her community has confronted him or her with another risk: that of getting lost, of losing one’s cultural homeland, that is, the reference system within which we feel at home and are able to act and communicate (De Martino, 2002). Ernesto De Martino’s thought comes to our rescue in explaining another fundamental aspect of the human condition: the individual’s commitment to operate according to intersubjective values through which to communicate with others is continually renewed as a need to overcome mere biological individuality, directing it at every moment toward the permanence of life “that counts”.

“The individual, who is man, is founded and maintained as such by this valorizing emergence of presence, by this unveiling of the private to the public, by this world of others in which one listens and responds, in a discourse that knows respite barely in dreamless restorative sleep (for even in nocturnal dreaming the discourse continues, albeit in a ciphered form for the awakened consciousness)” (De Martino, 2002, p. 264).

The sudden, lockdown-imposed loss of “worthwhile” life has caused a general sense of suspension and disorientation that has paralyzed entire communities, confronting them with the possibility of their own demise and enclosing them in the circumscribed spaces of the home (Ciampi, 2020). The Freudian formula of the Unheimlich seems formulated precisely to define this feeling of disruption, for it refers to everything that arouses fright, suspicion, and disquiet, insofar as it is unfamiliar, familiar, “of home.” It was precisely to the home (heim) that government authorities devolved the conservative and protective power, while individuals quickly adapted to the measures of confinement, concentrating in this place of high symbolic value new lifestyles and needs, new combinations of the public–private relationship, entirely new forms of work and leisure. The domestic microcosm, a fortress of privacy, has become a refuge and security, but also an arena of “struggle” between genders and generations, between individuals and households. Outside it, the unreal silence of the desertified urban space restored dystopian forms of places, freezing a fundamental aspect of living, in which the complex system of social relations comes to life. It can be said that the home has in a sense “boxed the world,” reduced it to a controllable dimension, displacing within it—within the domestic walls and in the virtual world—movements, sociality, conquest, wandering, and the path to a destination.

It was a change as silent as it was invasive and inexorable, affecting the places we inhabit and our bodies. We discovered ourselves, precisely because we were reclusive, deeply in need of citizenship, mentally and psychologically projected toward the outer space, which appeared all the more emptied of meaning and de-functionalized the more the inner-private space was re-functionalized. The home, which has always been a place-text to interrogate, one of the most powerful elements of integration of human thoughts, memories, and dreams, whose cohesive factor is represented by the reverie (Bachelard, 2006), was transformed on the phenomenological, functional, symbolic, and value levels.

Without losing its centrality, dwelling was thus reduced to immobile survival, the domestic topography was redesigned according to new needs as they emerged, modifying the spatiotemporal projection and altering the mobility of individuals (both in their somaticity and in their emotional and intellectual drives). That of the home is after all only a part of the larger experience of the world, which involves leaving home as a point of initiation into the outside world. The experience of this “other” space, which is the urban space, arising from the city and expanding from it to encompass all the places of social vitality, gives value to dwelling in the interior space, the domestic space.

In the pandemic, the relationship between these two fundamental spaces has almost been turned upside down, transforming the essence and distinctiveness of the space-home, usually perceived as a place capable of concentrating—in its own elemental ways—all the meaning of our existence in the world.

## 2 The ambivalence of domestic space

Although receding from everyday experience, the confinement phase has left a trace in our way of dwelling, understood in the deepest sense of being-in-the-world. Dwelling is a repeated event, a ceaseless flow that takes new directions each time. No one can teach us how to dwell, even though humanity has always inhabited this land, marking its history through the repetition of acts, the attribution of meanings, and the choice of design: despite the fact that it is a routine that unites everyone mutually, this remains a unique experience, where man performs gestures and activities as if each time were the first time.^2^

It can be asserted that in the modern way of living the community shares the experience of inhabiting a house, but that only the individual knows what it means to experience home. In fact, there is a fundamental distinction between the two terms: house refers to a physical, tangible, material reality, while home refers to a set of psychological, cultural, intangible meanings. Therefore home stands for the relationship that is established between house and its inhabitant, and house is the basis on which a home experience is developed. If the “center” of the dwelling experience is universal, natural, and affects everyone indiscriminately, its periphery, its infinite and multiple branches, make it the most personal and individual of human experiences. While the house is a concrete physical space whose expressive form is architectural design, the home is the practice (and process) of mediating between body, activity, and consciousness. The latter is also a social product and producer, since through the repetition of a taxonomic and generative pattern, the values of society are reinforced (Meschiari, 2018, p. 52).

Historical cycles and individual life cycles thus move in parallel along the living dimension, in a relationship between bodies and spaces (Merleau-Ponty, 2009), which involves an awareness, a feeling that only later turns into use, norms, and enjoyment.

Dwelling is thus both historicity and individuality: on the one hand, an expression of the position occupied in a given society and culture; on the other hand, a manifestation of a subject who, as a circumscribed, unique, and unmistakable individuality, is the only one capable of perfectly adhering to that dwelling, recognizing it as “theirs” and simultaneously recognizing themselves as “theirs.” Philosopher Maurizio Vitta reminds us that dwelling and habit have a strong connection starting from etymological evidence: habit is not a set of mechanical and boring acts, rather it is a psychophysical filter that allows us to absorb the surrounding space, endowing it with intimate signifiers through which it is constituted as our second nature. In essence, “dwelling is the place of the body, and the body is the place of repetition, of temporal cadences, of social rituality no less than of biological rituality” (Vitta, 2008, p. 227). Domestic existence, which is consumed in the protected enclosure of living, punctuates its time in obligatory steps, in stages that follow one another at constant intervals and presuppose the reiteration of gestures, actions, behaviors (Galimberti, 2009). If the human–space relationship is nothing other than dwelling thought of in its essence, we need to reflect on the new possibilities that living spaces can offer us, since “the space of dwelling, which coincides with that of the inhabitant’s body, not only identifies a position, a being-here, but also describes a situation, a way of being-for the rest of the world. In other words, it poses the problem of the inhabitant’s identity” (Vitta, 2008, p. 24).

Everybody has their own location, and the space of the body and the space of the world are consonant, touching, and blurring. But the body, as Michel Serres notes, does not “operate” in a single spatial dimension, but rather a varied one, the result of intersections and topological correlations. The scholar refers to an epistemological model of qualitative analysis of the spatial varieties in which we live as organic, cultural, and social bodies, and hypothesizes that each culture constructs precise ways of connecting these families of spaces. The idea of the body lowered into a space that is not unique returns—further deepened—in some of the examples given by Serres: in Euclidean space, the body works, but it only works there; in projective space, it sees; in one topological variety, it touches, caresses, and handles; it feels and communicates in another. The body, then, is dropped into the intersection of a numerous family of spaces and this intersection is not “given,” “these connections are always to be constructed” (Serres, 1986, p. 30).

The body inhabits as many spaces as those formed by the society, the group, the community: the home, the streets to be walked, the garden, the churches, the school, the places of worship, and the secular ones. Space is a frequented place, an intersection of many mobilities, which, for example, transform the street geometrically defined as a place of urbanism into space. It follows that the very relationship between individuals and their places of dwelling embodies the characters of subjectivity, dynamism, and temporality.

Subjectivity, in that the individual is not an abstract self, but cast in a very specific existential condition. They are a subject-synthesis of their own singular experience (habits, thoughts, ways of being) and of their social-historical experience (taste, “mentality,” collective way of being and feeling): changing the latter, through the centuries, changes the phenomenology of the values of the “internal” space and, therefore, the stylistics of the built forms. Therefore, the individual enlarges the personal and particular way of being, dilates it, and relates it to the feelings and dimensions of the historical time in which they live.

Dynamism, in that subjects and objects, while forming a “whole,” in their mutual connection constitute a continuous interplay of cross-references, in which the individual thought stream comes into contact with things, perceives them, registers the differences, and returns to the mind, provoking a definite attitude and sensation. Gregory Bateson speaks of a relationship of contemplation, of perceptions that can cause pleasantness or dissatisfaction, because the place and surrounding objects are the terminal of the self, recipients capable of establishing with the subject a continuous flow of sensations (Bateson, 1972). It is for this reason that if a stranger violates the domestic space, one feels attacked in one’s intimacy and experiences a feeling of panic, of defilement.

Temporality, finally, as a dimension that acts on place and, in this change, involves the subject by reconnecting him or her to the history of his or her time. Dwelling is thus a condensation of individual and collective experience, of mental and historical structure.

The intimate relationship between the three dimensions makes dwelling a complex but essential condition for humans because it pertains to their way of being on earth. To be in the world is to have a world, understood not only as a host place, but above all as a term in which to project oneself, something that has to do with intentionality and projectuality. Martin Heidegger’s thought is illuminating in this perspective because it recognizes the intimate relationship between being and inhabiting: “To be man means to be on earth as mortal, namely: to inhabit” (Heidegger, 1976, p. 97). In this sense, the philosopher operates an extension in essential terms and not a limitation by approaching it with the meaning “of being.” More specifically, the essence of dwelling is linked to the concept of being content, having peace, being free, protected from evils and threats: “the fundamental trait of dwelling is this having care (Schonen)” (Ivi, p. 99).

An interesting feature of the Heideggerian analysis—when related to the experience of the pandemic—is found in the analysis of the relationship between humans and space: space is not something that is in front of humans; it is neither an external object nor an inner experience. There are no humans and “also” space. Even when we relate to things that are physically unreachable, we still stay with the things themselves. According to Heidegger, it is not true that we simply inwardly represent distant things to ourselves in such a way that, instead of these, only representations parade in our intimacy and in our heads. If we think, for example, of the old Heidelberg Bridge, even at a distance we are there at the bridge, and not instead at some representative content of our consciousness. Indeed, even by being far away we can be, with respect to that bridge, much closer than a person who crosses it daily as any thoroughfare. The experience of dwelling does not consist, then, only in the relationship of proximity to places and objects: thinking is indispensable to dwelling, since for a human to be is to be always in a situation planning to change it, since life is essentially made up of needs, desires, interests, affective states, which are all ways of leaning toward the future. The very spaces that we normally take for granted, those of daily living and social encounters during the lockdown have been revealed as essential, inalienable, and irreplaceable. For the same reason, human existence as a project, as a projection toward the other, in a dynamic and relational relationship cannot abruptly change.

With reference to dwelling, the contribution of Emmanuel Lévinas appears to be even more focused on the theme of home: the subject really finds himself to the extent that he is at home, both in the sense of possessing a place with respect to which the world is configured and in the sense of having a place to take and store what the world offers at hand. Within the world, home belongs to the paraphernalia of things indispensable to human existence; it is necessary as protection, nest, but also as a property of enjoyment. However, it is not only necessary as an object of use among many “usable” ones, for it occupies a pregnant role in the system of purposes in which human life is placed. It is from the house that man’s existence begins; the privileged role of the house consists not in being the end of human activity, but in being its condition and, in this sense, its beginning. People situate themselves in the world as moving toward it from a property, from a home into which they can, at any moment, withdraw. Simultaneously outside and inside, people situate themselves outside starting from an intimacy (Lévinas, 2006). But also our internal living, as Franco La Cecla explains, is always a ‘living of’, i.e. it needs external things: the city, the world, and is satisfied with them or misses them (La Cecla, 1993). Everything begins in the place-home where one gathers, creating a separation with nature and the outside. To be separate, according to the French philosopher, means to dwell somewhere: separation is positively produced in location. From this property and its inherent intimacy, people place themselves toward the outside. Lévinas thus emphasizes that it is the world that situates itself with respect to dwelling and not the other way around. In this sense, the house stands as the heart of the symbiotic relationship between the individual and his spatial surroundings, as a place most invested with symbolic value, but also a metaphor for a ‘fixity’ that attempts to give dynamically order to the speed, acceleration and liquidity of ‘external’ living.

These reflections constitute a starting point for questioning ourselves about the changes imposed by the pandemic and the “disorder” it has generated in our way of dwelling: it is undeniable that the initial “hyper-habitational” obligation of confinement has risked undoing the correctness of the relationship between the ego and things, between the body and the objects that animate its space. The ritual of “returning home” constitutes a healthy and satisfying transition as long as, and provided that, it coincides with the re-appropriation of intimate space and is normally abandoned for public, external, social space. The boundary of the domestic threshold has the sense of an Edenic introitus only if the body can resume self-care in the warmth of habits, ridding itself of the social “artifices” and cultural masks that daily enable it to maintain lasting relations with the external environment. It has been said that living is an active, moving, dynamic experience, so it is always a plunging into a dense, subtle fluid, made up of routine gestures, of sensations through which the dialectic between inside and outside is reproduced each time.

The pandemic has converted the space of freedom and domestic family intimacy into something totally unprecedented on the ritual and experiential level and difficult to assimilate on the psychological level. It has redesigned and mentally affected the sense of living and its semantic complexity, having imposed the home as the primary space, both as a defense against health risk and as an ergonomic place. To this day, the domestic space takes on and absorbs “other” functions that were not its domain before (e.g., smart working, digital sociability, and sports activity); homes have opened up to new uses, becoming places more susceptible to modification and rethinking, in the wake of habits that were somehow introjected even if initially dictated by the state of uncertainty and vulnerability.^3^ Over the past three years, between epidemic peaks and recovery of normality, we have witnessed a “fracture of the home” or rather “deviant” behaviors with respect to domestic rituals and the canonical functions of space and time: definition of the roles of individual rooms, family liturgies, more or less strict schedules, respect for privacy, worship of objects, etc. The home has to some extent curbed the disorder generated by the fear of the pandemic, but the world has burst powerfully into the domestic sphere, thanks to increasingly sophisticated and fast technological and informational means, facilitating greater contact with external reality and a continuation on the social, educational, and work levels. Dwellings have seemed to be house-hiding places, but at the same time they have been configured as open houses: it could be asserted, with the help of mythological figures, that the phenomenology of living space during COVID-19 has visibly changed due to the triumph of Hermes over Hestía^4^ (Augé, 1992).

Even today, after such a devastating pandemic, there is confirmation of the relevance of the Heideggerian discourse: dwelling resists as a synonym for protection, peace, freedom, since the latter always presupposes security, and security is possible only through subjective identity, of which existential space constitutes the foundational aspect.

But the lived experience of communities has left visible traces in the times of life, and thus also in those attributed to living: after the stasis and deceleration of daily rhythms, the temporal cadence of every activity has recovered the speed of the pre-pandemic period, even in domestic interior spaces, with a tendency toward acceleration and the “regaining of lost time.” Technology is ready to correspond to the needs induced by the outdated fear of COVID-19 and stimulates them by offering the means and tools of speeding up: it is the triumph of smartness as an adaptation to the new and as an increasingly widespread model. On this prospective horizon, which will require new analyses, including sociological ones, the smart home is already a reality, the result of a theoretical–practical approach to contemporary demand. However, the positive aspects that go toward modernization will have to be combined with standards of balance and control of technological resources, in accordance with an idea of progress that does not dehumanize the meaning-even symbolic-of living.

By now, we can see quite clearly (…) that the prospects that open are at least as rich as those that close, that we will be able to live in dilated dimensions (…), that the humanity that will develop in a world of extra-familial relations of extra-national cultures, of extra-religious morals will be—I do not say better or worse than before, which makes no sense—but it will be varied, different, complicated, meaningful, with values, not insulting, happy-unhappy, in short it will be (Calvino, 1995 pp. 106–107).

## 3 The smart domestic space

The pandemic theme has highlighted how much the symbolic and relational dimension of living has been shared with the dimension of smartness. A necessary prerequisite in this regard is to define what smartness is and does. Leaving aside the definitional issues that already animate the scientific debate on the issues, it is worth immediately pointing out how two strongly interrelated dimensions appear among the axes of this conceptual typology: the technological dimension and the dimension of sustainability (Hollands, 2008). In this direction, the smart home can be defined as the product that, more than any other, frames smartness. The smart home is defined by some as the technological home (Giffinger et al., 2008) and by others as the ‘sustainable’ and zero-emission home (Moraci and Fazia, 2013). There are also those, however, who not only analyze its externalities, but also consider its internal functionalities, hence the smart home is defined as “adaptive,” “sensory,” and “attentive” (Pantzar and Shove, 2017; Lupton, 2018). It is precisely those same relationships that have made the smart home a great ally of the community during the pandemic period. The role of the “smart” home became apparent in all its importance, since the confinement due to the restrictive measures adopted, at different times, by all countries, put housing, and living altogether, back at the center of life and sociological debate.

Thus, homes that were smart, sustainable, technological, adaptive, and above all interconnected withstood the shock of containment, the overcrowding of their spaces. Homes that lacked smart designs, on the other hand, had to “chase” necessity. The absence of physical connection has, by contrast, demanded the present and constant interconnection of all social apparatuses, and has made visible, as never before that moment, how the smart society is not a destination to strive for, but is, in some ways, a necessary solution. It was not, in this sense, a matter of espousing one or the other faction (Eco, 1984; Iannone, 2007) because it is not a matter of reasoning about the presence or not, the use or not, of technology. Instead, it is a matter of recognizing in the smart home a project that is technological, efficient, sustainable, and that makes these facets dialog in one grand narrative. During the pandemic period, the interconnection promoted and advanced by the smart home resulted in disconnected forms of connection. Interconnection on the technological and digital level has not been followed by physical and proximity connection. Home-alone services, i.e., those services that potentially allow the individual to remain inside his or her apartment without interacting with others, have increased. That is, they represent new realities that lead the individual to isolate themself, thus defining a new way of being together and responding to the need for relationship: a being together while being physically confined to one’s living space.

It is in this sense that the smart home should not be seen from the outside as only a technological home, but that it should be included precisely by virtue of the above, in a systemic perspective. The health emergency has not only brought back to the center of attention the role of the home, but also, with equal power, the role of the semi-private and private spaces that relate to it. In this direction, the smart home has been hit by a powerful revolution in meaning that has allowed its role to be reinterpreted in a direction of total sustainability. The smart home can indeed be said to be sustainable in that, in a changed social context (especially since the pandemic), it seems to have the ability to use smart technologies to reinterpret society, including understanding how they had to adapt.

In this direction, the ideal smart home is embedded in a context that rediscovers proximity around the home as an added value. The violence of the effects of the pandemic, in addition to crippling the lives of entire communities, has broken the elasticity and dynamism that characterize social life (Simmel, 1998), disrupting the very concept of dwelling and the emotional disposition of “feeling at home” (Vitta, 2008). These are not exhausted in the domestic sphere, but are nurtured by living in places that are other, filling public spaces and animating the world of objects through everyday practices, customs, and experiences of an exterior whose very definition is related to an interior (Ciampi, 2011).

It is not only understood as a proximity in terms of a “proximity house” but instead precisely as an “augmented city,” keeping in mind that proximity is not only physical, such as private proximity in the pandemic context, but is a relational proximity. The idea of “augmented cities” is in fact founded on a new ideology that reduces centripetal mobility by ensuring easy access to places: a new ideology that bases its development on the new paradigm “people, planet, profit” (Kotler et al., 2010) that seems to well represent the aspiration that affects the social and economic organization of cities. The creation of the new city models cannot be developed without a revisiting of the city’s networks, which implies taking into consideration urban planning and service design choices that connect economic and social living aspects. Large metropolises tend to empty out in a kind of new repopulation that sees the countryside as the protagonist; on the other hand, cities undertake projects in which proximity is added value and leverage for other innovations suitable for tracing policy agendas related to the goals of Agenda 2030 (Mangini, 2020).

The pivotal post-pandemic principle on which the placement of the smart home as a “project” should be based is then the concept of “staying near one’s home.” In the systemic perspective that suggests the direction of proximity, one could rethink common and urban places, including, in these vicinities, social activities that implement collective relationality such as collective gardens, productive activities, and places for safer social relations. It comes, in this sense, and thanks to smartness, to define an “augmented domesticity” of public space in which not only individual but also collective, social, and economic relations activities are possible, which, starting from the house, expand into the city.

Precisely in relation to the house inserted into a system, of places as well as of people, there are also those who have thought of creating actions so that this systematicity inserted on paper would also be a reality. In this direction, a neighborhood concierge or even services for the person have been thought of that include formulas at reduced prices of six-month or annual subscriptions for parcel pickup, coworking spaces, key storage, bike repair, or dog sitting activities. These are just examples one can think of when thinking of proximity facilities. The rediscovery, during quarantine days, of courtyards and proximity facilities, as opposed to our living quarters, has also determined an important value for mental and physical well-being (Porcelloni and Mazzanti, 2020). The roofs of buildings have turned into gardens where we can sunbathe, squares where we can play, or terraces where we can exercise while remaining within the domestic space and expanding the natural landscape of the city. The possibility of living in a happy city would seem, then, to be achieved by starting from the idea of exploiting all the spaces of the territory itself, fostering cohesion and the social dimension, starting from the small cores of society and then expanding to the whole city by taking advantage of every technological outcome.

In recent years, and especially during the pandemic period, the idea of trying to contain travel within the city to minutes has then been launched, since space and time represent a strongly incisive combination for lifestyles and consumption for people. The examples are varied: Melbourne has launched the “twenty minutes neighborhood” plan; Copenhagen has a neighborhood dubbed “five minutes to everything”; even Paris has appointed an alderman to the “City of the Quarter Hour,” aiming for an urbanism in which everything is no more than 15 min by foot or bicycle (Hausmann et al., 2020; Rinnovabili.it., 2020). These are policies that have been on the agenda for quite some time, but with the emergencies related to COVID-19, they have received the push needed for their effective implementation.

Tying, then, travel and relationality to a reduced time does not only mean decreasing the distant spaces, but it also means, precisely in relation to these displacements, focusing on an ecological vision that can provide new stimuli to the social life of the neighborhoods by fostering cohesion among the people who live in them. Everything is quickly accessible, on foot, by bicycle, and from the smart home we move to the broader smart district dimension. The hyperplaces that have flourished in recent years “must layer with the body of the city made up of people and the architecture, which is not sculpture of the city, but a frame of sense of relationships” (Mangini, 2020).

## 4 The smart home of the future

In light of the renewed relationship between housing and neighborhoods, the legitimate question to ask is how the smart home of the future will be, especially in relation to the new needs of proximity. Whether it will be sustainable, in the terms dictated by the Brundtland Report (United Nations, 1987; World Commission on Environment and Development, 1987), or whether it will continue to be an idea on paper without any real implementation. Certainly these are just some of the doubts inherent in the realistic feasibility of the smart building process. In fact, as Rifkin (2022) reminds us, smartness is assigned the role of “generating” efficiency. However, an efficiency that becomes the instigator of profoundly new lifestyles. This rethinking of everyday life and this reimagining of reality can, not hardly, be traced back to the dichotomy that Beck (2016) presents between change and metamorphosis considering processes belonging to the field of metamorphosis as processes of more complicated management.

As seen, it is a matter of rethinking the house not only as a separate unit but rather as an adaptive and proximate house, in other words, a sustainable smart home in all aspects of sustainability, not just environmental. It is precisely the interrelationships between all sectors of sustainability that allow the smart home (and smartness itself) to present itself as a solution and objective, among others, of the Sustainable development goals (SDGs), although, as the scientific literature well shows, the aspect of sustainability related to the environment seems to be taken more into consideration.

Towards greater collaboration between all facets of sustainability, the house, as well as the surrounding space, seem to belong to three areas of action (which will be called cycles) that involve the social actor and derive from the integration of different sustainability models (Agazzi et al., 2020). Models that demonstrate how the facets of sustainability must continue in parallel and not, instead, allow one to prevail over the other.

The first cycle is that of attraction, a purely economic cycle in which the promise of investors is developed and which sees environmental and social sustainability give way to economic sustainability. The (economic) increase of the first cycle leads to the second cycle, that of well-being, which thrives as long as the expansive phase seems to produce benefits equally. The third and last cycle is that of gentrification, which produces an increase in inequalities and expulsion from the city following speculative actions and which sees social sustainability crack. The pandemic phenomenon has inevitably illuminated this cycle related to inequalities, as it has made citizens more fragile and has urgently required the implementation of alternatives that adopt survival techniques even before widespread well-being. The priorities of groups of citizens are on the taut ropes between the extremes of autonomy and dependence (on the x-axis) and trust and disenchantment (on the y-axis).

So it is in this direction that the construction of the smart home should go. At present, it is only a model that holds up in theory, especially in terms of sustainability. Defining the boundaries of sustainability when it comes to housing is not enough; practical sustainability must also have practical implications in reality. For example, the house should not only be a hub connected to the internet, but also an interconnected space with all the other neighborhood systems, or it should be low-cost, allowing disadvantaged social groups to purchase it. It should also be environmentally sustainable.

The idea is that the rules of living and building design, neighborhoods, and condominiums are changing in the wake of the pandemic. In practical terms, the team “Design Force 8,” consisting of numerous Italian and international architecture and design studios, claims that buildings designed after COVID-19 can be disassembled rather than demolished once they have aged, as they will be made of entirely recyclable modules according to the rules of the circular economy (Ansa, 2020).

What the COVID-19 pandemic has shown concerns mainly how homes are lived in internally and how internal relationships must change to face the aftermath of COVID-19 and its consequences by learning to live with the virus. Houses have represented an effective response against infection, determining the structure and form of the buildings in response to the infection.

For example, there has been a shift from the demand for domestic greenery during the pandemic (with consequent smart devices for plant growth and nutrition) to the design of urban greenery in the vicinity of housing areas. The reasoning that has necessarily stemmed from homes due to COVID-19 is reflected in constructions that play on the internal/external binomial with respect to the surrounding social space (Kretchmer, 2020). This, in turn, influences the design of roads, transport networks, buildings, and internal and external environments. In this sense, there is a shift from private, internal awareness and design to external proximity, and the consequent construction of intelligent environments, if the starting point is the smart home.

However, building intelligent environments means building assistive environments that become enabling, especially in terms of sustainability. In this sense, the problem of housing sustainability should contemplate two possibilities: not only should sustainable performance be considered (e.g., a house that does not pollute with its emissions) but sustainable design as well (in terms of materials, geothermal impact, social impact, etc.). A smart sustainability that is “by design” as well as “by default,” echoing a dichotomy already used by the European Union in its designs. Only in this sense do smart technologies that are already being designed considering sensory, cognitive, psychosocial, and emotional characteristics extend their range of action—and therefore design—when they must be incorporated into the domestic environment.

Sustainability, therefore, materializes mainly from the design phase. It is in this direction that the question “what makes sense to do?” becomes necessary when designing products and equipment so as to decrease the probability that they will be abandoned because they are not sustainable in terms of usability. The need to consider “instructions and manuals, legislation and regulatory standards, cultural context, and aesthetics” tells us a lot about the systematic nature of these technologies and the difficulty of implementing and contributing to change (Stuto, 2022).

In addition to this, the challenge of the smart home is not only in reconciling multiple aspects of sustainability to reflect them on nearby environments, but rather in that of involvement and full participation in the built environment, a challenge that puts the social actor and their relationship with the surrounding environment, both internal and external, back at the center.

Sustainability and system, therefore, are two terms that travel together, both because sustainability is systemic and because an entity must allow “present generations to meet their needs without compromising the ability of future generations to meet their own,” as stated in the Brundtland Report (U.N. 1987), to interact in an orderly and organized manner. A system understood as infrastructure supports communities of all kinds.

The goal, then, does not seem to be to design “the house of the future” in the name of the smart home (Bilò and Palma, 2020), but to find in the living space the cues to rethink a design that is both functional and respectful of the real needs of humans and communal living.

## Author contributions

All authors listed have made a substantial, direct, and intellectual contribution to the work and approved it for publication.

## Footnotes

^1^On the other hand, contemporary society is a highly complex, composite, multidimensional, and reflexive system and, as such, has more numerous variables and higher risk components. According to Niklas Luhmann, conditions of risk, that is, of the unpredictability of events (or some events), are normal qualities of the system, since any complex society is itself inevitably risky, even when it strives to function according to seemingly rational rules (Luhmann, 1996).

^2^In this regard, it is interesting to recall, on the level of a symbolic reconstruction, the dream of the multi-storey house recounted by Carl G. Jung in his memoirs. The house, with its storeys, symbolically represented all the stratifications of his individual consciousness, right down to the most remote layers of the ‘collective unconscious’ (Jung 1983).

^3^These very premises are the basis for the empirical investigation coordinated by the writer, as principal investigator, as part of the National Research Project “Inhabiting uncertainty. A multifaceted study on the relationship between social attitudes and lifestyles in pandemic spaces” (2020). The research investigates, through mixed methods, the transformations of the post-pandemic housing experience experienced by the youth population of secondary schools (pertaining to the Rome context) and young people attending the first two years of university. The study analyzes the following dimensions: spatial (home, urban context) and temporal; relational, social, and community (online and offline); emotional/value; and of imagination and creativity (leisure and interests, expectations, and perception of the future).

^4^Augé observes that Hestía, goddess of the domestic hearth and protector of family peace and prosperity, symbolizes the fulcrum of the home, the closed and intimate space of the group gathered about itself, and, in some ways, also the relationship with oneself. In contrast, Hermes, god of inventions, trade, roads, and pastures, but also of the threshold and the door, depicts movement, relationship with others, and the entrances to cities. Identity and relationship constitute, for the French anthropologist, the center of all spatial systems, including the home, classically analyzed by anthropology.

## References

[ref1] Agazzi D. Brambilla M. Daelli S. (2020). Città dal futuro, Crediti Città dal futuro, 9 Novembre 2020. Available at: https://cittadalfuturo.com/ (Accessed June 20, 2023].

[ref2] Ansa (2020). Abitare, c'è un prima e dopo covid-19. Le soluzioni dei designer. Available at: https://www.ansa.it/canale_lifestyle/notizie/design_giardino/2020/05/20/abitare-ce-un-prima-e-dopo-covid-19.-le-soluzioni-dei-designer_93bde3da-13a5-42f2-a131-7ae86ad5d0da.html (Accessed June 20, 2023].

[ref3] Augé M. (1992). Non-lieux. Paris: Éditions du Seuil.

[ref4] Bachelard G. (2006). La poetica dello spazio. Bari: Dedalo.

[ref5] Bateson G. (1972). Steps to an ecology of mind. San Francisco: Chandler Publishing Co.

[ref6] Bauman Z. (2014). La vita tra reale e virtuale. Milano: EGEA.

[ref7] Beck U. (1992). Risk society: Towards a new modernity. London: SAGE Publications Ltd.

[ref8] Beck U. (2016). The Metamorphosis of the World: How Climate Change is Transforming Our Concept of the World. Cambridge: Polity Press.

[ref10] Bilò F. Palma R. (2020). “Il cielo in trentatrè stanze” in Cronache di architetti #restatiacasa (Siracusa: Lettera Ventidue).

[ref11] Boccia Artieri G. Gemini L. Pasquali F. Carlo S. Farci M. Pedroni M., (2017). Fenomenologia dei social network. Presenza, relazioni e consumi mediali degli italiani online. Milano: Guerini.

[ref12] Bontempi E. Vergalli S. Squazzoni F. (2020). Understanding COVID-19 diffusion requires an interdisciplinary, multi-dimensional approach. Environ. Res. 188:109814. doi: 10.1016/j.envres.2020.109814, PMID: 32544726 PMC7289085

[ref13] Calvino I. (1995). “La sfida al labirinto” in Una pietra sopra (Milano: Arnoldo Mondadori Editore), 99–117.

[ref14] Ciampi M. (2011). Forme dell’abitare. Un’analisi sociologica dello spazio borghese. Soveria Mannelli: Rubbettino.

[ref15] Ciampi M. (2020). Profili sociali e immagini dell’emergenza sanitaria. Rivista Trimestrale di Scienza dell’amministrazione. Studi di teoria e ricerca sociale 2:2020, 1–30.

[ref16] De Martino E. (2002). La fine del mondo. Contributo all’analisi delle apocalissi culturali. Torino: Einaudi.

[ref17] Eco U. (1984). Apocalittici e integrati. Milano: Bompiani.

[ref18] Esposito R. (2022). Immunità comune. Torino: Einaudi.

[ref19] Favretto A. Maturo A. Tomelleri S. (2021). L’impatto sociale del Covid-19. Milano: FrancoAngeli.

[ref20] Fuchs C. (2020). Everyday life and everyday communication in coronavirus capitalism. TripleC 18, 375–398. doi: 10.31269/triplec.v18i1.1167

[ref21] Galantino M. G. (2020). Tra pandemie annunciate e vere pandemie: dalla SARS alla COVID-19. Rivista Trimestrale di Scienza dell’amministrazione. Studi di teoria e ricerca sociale 2 2020, 1–27.

[ref22] Galimberti U. (2009). Il corpo. Milano: Feltrinelli.

[ref23] Giaccardi C. Magatti M. (2020). “Nella fine è l’inizio” in In che mondo vivremo (Bologna: il Mulino).

[ref25] Giddens A. (1990). The consequences of modernity. Stanford: Stanford University Press.

[ref26] Giffinger R. Fertner C. Karmar H. Kalasek R. Pichler-Milanovic N. Meijers E. (2008). Smart cities: Ranking of European medium sized cities. Center of Regional Science, Vienna University of technology. Vienna University of Technology. Available at: http://www.smart-cities.eu (Accessed June 20, 2020).

[ref27] Habermas H. (2022). Proteggere la vita. I diritti fondamentali alla prova della pandemia. Bologna: il Mulino.

[ref06] Hausmann C. Camera A. Terribile V. (2020). Il futuro delle metropoli, la città del quarto d’ora. Rinnovabili.it.

[ref28] Heidegger M. (1976). Saggi e discorsi. Milano: Mursia.

[ref29] Hollands R. (2008). Will the real Smart City please stand up? City 12, 303–320. doi: 10.1080/13604810802479126

[ref30] Iannone R. (2007). Società dis-connesse. La sfida del digital divide. Roma: Armando.

[ref01] Jung C. G. (1983). Memories, Dreams, Reflections. London: Flamingo.

[ref54] Kretchmer H. (2020). COVID-19: Is this what the office of the future will look like? World economic forum.

[ref31] Kotler P. Kartajaya H. Setiawan I. (2010). Marketing 3.0: from products to customers to the human Spirit. Hoboken (NJ): John Wiley & Sons.

[ref02] La Cecla F. (1993). Mente locale. Per un’antropologia dell’abitare. Milano: Elèuthera.

[ref32] Lévinas E. (2006). Totalità e infinito. Saggio sull’esteriorità. Milano: Jaca Book.

[ref33] Luhmann N. (1996). Sociologia del rischio. Milano: Mondadori.

[ref34] Luhmann N. (2002). La fiducia. Bologna: il Mulino.

[ref35] Lupton D. (2018). Smart homes and their users. Oxon: Routledge.

[ref36] Mackenbach J. P. (2019). Health inequalities: Persistence and change in European welfare states. Oxford: Oxford University Press.

[ref37] Mangini D. (2020). Genova come Copenaghen: così il sogno delle “città del quarto d’ora” rimodellerà l’economia. Forbes Available at: https://forbes.it/2020/12/07/citta-del-quarto-d-ora-rimodella-economia/ (Accessed June 20, 2023).

[ref38] McNeill W. H. (2020). La peste nella storia. Milano: Res Gestae.

[ref39] Merleau-Ponty M. (2009). Fenomenologia della percezione. Milano: Bompiani.

[ref40] Meschiari M. (2018). Disabitare. Milano: Meltemi.

[ref41] Moraci F. Fazia C. (2013). The smart cities and challenges of sustainability. TEMA J. Land Use Mobil. Environ. 6, 35–45.

[ref42] Morin E. (2020), Cambiamo strada. Le 15 lezioni del coronavirus. Milano: Raffaello Corina Editore.

[ref43] Pantzar M. Shove E. (2017). Home extensions: rethinking the smart home through social practice theory. Sci. Technol. Stud. 30, 50–66.

[ref44] Porcelloni L. Mazzanti C. (2020). Spazio sicuro e non-sicuro: un’indagine sulle nuove strategie dell’abitare nel contesto della pandemia di Covid-19. Documenti Geografici 1, 633–646.

[ref03] Rifkin J. (2022). The Age of Resilience. Reimaging Existence in the Rewilding Earth. London: Swift Press.

[ref45] Rinnovabili.it. (2020). Il futuro delle metropoli: Il modello "della città del quarto d'ora". Available at: https://www.rinnovabili.it/greenbuilding/smart-city/citta-del-quarto-d-ora/ (Accessed June 20, 2023).

[ref46] Sassen S. (2007). A sociology of globalization. W. W. New York: Norton & Company.

[ref47] Sassen S. (2014). Expulsions. Brutality and complexity in the global economy. Cambridge (MA), London: Belknap Press.

[ref48] Serres M. (1986). “Discorso e percorso” in L’identità. ed. Lévi-Strauss C. (Palermo: Sellerio), 25–49.

[ref49] Simmel G. H. (1998). Sociologia. Milano: Comunità.

[ref04] Stuto S. (2022). Costruire Ambienti Intelligenti significa costruire Ambienti Assistivi. Con le tecnologie questo è possibile. Tech Economy 2030.

[ref51] United Nations (1987). Our common future. Report of the world commission on environment and development. Available at: https://sustainabledevelopment.un.org/content/documents/5987our-common-future.pdf (Accessed June 20, 2023).

[ref52] Vitta M. (2008). Dell’abitare. Corpi, spazi, oggetti, immagini. Torino: Einaudi.

[ref53] Viviani L. (2021). Mito e realtà dell’impatto della pandemia su società e politica globali. Note per la ricerca sociale. Società Mutamento Politica 12, 179–183.

[ref05] World Commission on Environment and Development. (1987). Report of the World Commission on Environment and Development: Our Common Future.

[ref55] Wright Mills C. (1959). The Sociological Immagination. Oxford: Oxford University Press.

